# A Dynamic Analysis of Tuberculosis Dissemination to Improve Control and Surveillance

**DOI:** 10.1371/journal.pone.0014140

**Published:** 2010-11-30

**Authors:** Rita M. Zorzenon dos Santos, Ana Amador, Wayner V. de Souza, Maria Fatima P. M. de Albuquerque, Silvina Ponce Dawson, Antonio Ruffino-Netto, Carlos R. Zárate-Bladés, Celio L. Silva

**Affiliations:** 1 Departamento de Física, Universidade Federal de Pernambuco, Cidade Universitária, Recife, Pernambuco, Brasil; 2 Departamento de Física, FCEN-UBA, Universidad de Buenos Aires, Buenos Aires, Argentina; 3 Centro de Pesquisa Ageu Magalhães, FIOCRUZ, Recife, Pernambuco, Brasil; 4 Departamento de Medicina Clínica, Universidade Federal de Pernambuco, Recife, Pernambuco, Brasil; 5 Departamento de Medicina Social, Faculdade de Medicina de Ribeirão Preto, Universidade de São Paulo, Ribeirão Preto, São Paulo, Brasil; 6 Núcleo de Pesquisas em Tuberculose, Departamento de Bioquímica e Imunologia, Faculdade de Medicina de Ribeirão Preto, Universidade de São Paulo, Ribeirão Preto, São Paulo, Brasil; Institute of Infectious Diseases and Molecular Medicine, South Africa

## Abstract

**Background:**

Detailed analysis of the dynamic interactions among biological, environmental, social, and economic factors that favour the spread of certain diseases is extremely useful for designing effective control strategies. Diseases like tuberculosis that kills somebody every 15 seconds in the world, require methods that take into account the disease dynamics to design truly efficient control and surveillance strategies. The usual and well established statistical approaches provide insights into the cause-effect relationships that favour disease transmission but they only estimate risk areas, spatial or temporal trends. Here we introduce a novel approach that allows figuring out the dynamical behaviour of the disease spreading. This information can subsequently be used to validate mathematical models of the dissemination process from which the underlying mechanisms that are responsible for this spreading could be inferred.

**Methodology/Principal Findings:**

The method presented here is based on the analysis of the spread of tuberculosis in a Brazilian endemic city during five consecutive years. The detailed analysis of the spatio-temporal correlation of the yearly geo-referenced data, using different characteristic times of the disease evolution, allowed us to trace the temporal path of the aetiological agent, to locate the sources of infection, and to characterize the dynamics of disease spreading. Consequently, the method also allowed for the identification of socio-economic factors that influence the process.

**Conclusions/Significance:**

The information obtained can contribute to more effective budget allocation, drug distribution and recruitment of human skilled resources, as well as guiding the design of vaccination programs. We propose that this novel strategy can also be applied to the evaluation of other diseases as well as other social processes.

## Introduction

Despite the implementation of various control and surveillance strategies, infectious diseases remain among the leading causes of worldwide morbidity and mortality [1], [2]. For certain diseases, such as tuberculosis (TB), the number of newly infected people is rising steadily in certain areas [3]. According to the World Health Organization, WHO [3], there are approximately 9 million new reported cases of TB and 1.7 million TB-related deaths each year. Of the total number of TB cases worldwide, 80% are concentrated in 22 nations, including Brazil, known as high-burden countries [3]. It is of the utmost importance for countries such as Brazil to develop novel strategies to tackle the problem. In order to achieve the objectives of the WHO-Stop TB Program [3], these strategies should include not only a reliable system of information and an efficient method for localizing sources of infection, but also an enhanced understanding of the dynamics of disease spreading [2], [4]. Technologies such as remote sensing and Geographical Information Systems (GIS) have improved the reliability of spatial data related to infectious diseases, but these require new means of analysis, which have the capacity to yield information on the disease's dissemination dynamics [4], [5].

To date, most statistical approaches have estimated spatial or temporal trends using stationary probability density functions [6]–[9] and spatial models [10], which provide insights into the cause-effect relationships favouring disease transmission. However, analyses that do not take into account the dynamics of these processes have had limited influence on the design of strategies for controlling disease transmission and conducting surveillance [4], [10]–[12]. The novel approach presented in this study does not rely on stationary assumptions, but aims to use currently available data more effectively in order to design efficient control and surveillance strategies. Using information on disease dynamics the method pinpoints the sources of infection by signalling out regions that persistently report new TB cases; it can be of help to model the disease spreading and, in this way, gets an insight on the mechanisms underlying the process and ultimately it allows identifying the social factors involved in the endemic process. The results that emerge from this analysis will make it possible to develop new strategies for reducing local TB incidence and mortality in high-burden places. Finally, this method can be successfully transposed to the control and surveillance of a variety of other diseases and social processes as well.

## Results

**Figure 1:**
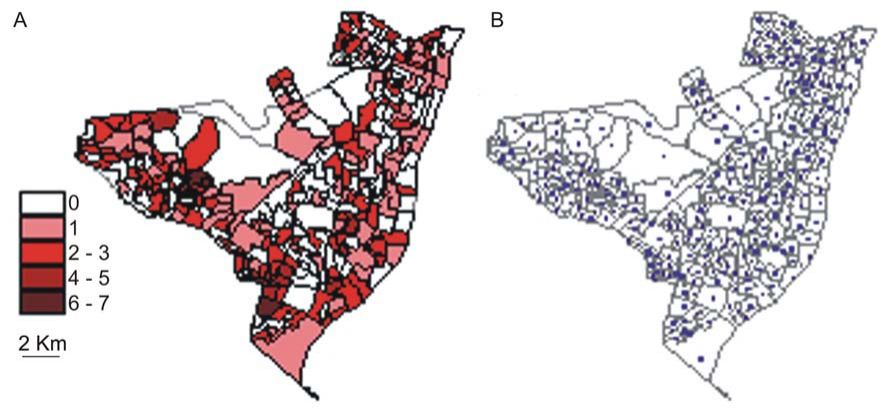
Olinda CT division and its geometrical centres. (A) Distribution of accumulated TB cases per census tract (CT) during the year 1996. The numbers associated with the coloured boxes represent the number of cases per CT. The map shows the polygons representing the census tracts (CT) into which the city of Olinda is divided. Each CT is an administrative district encompassing an average number of 300 households, or 1200 people. (B) Dots represent the geometrical centres of the polygons of the CT division (299 units) in the 2000 Census, in which we base our analysis of the annual distributions of TB cases for the 1996–2000 period.

We used this method to analyze the dynamics of the dissemination of TB in Olinda, a town of 370,000 inhabitants in the northeast of Brazil, where the incidence and mortality rates are higher than the Brazilian average [13]. These high rates reflect, among other factors like poverty and low schooling, the difficulties of the inhabitants to access the public health system where TB cases are diagnosed and treated. The data [14] recorded in Olinda constitute a five-year (1996–2000) data set of cases of pulmonary TB reported monthly, which are annually geo-referenced to the 299 local census tracts (CTs, see methods) from the 2000 Brazilian Census [15]. The TB cases are geo-referenced to the CTs according to the patient household and its geographical centre represents each CT. In Figure 1A, we show the distribution of CTs, together with the numbers of newly reported TB cases in 1996. Similar patterns of distribution per CT were observed for all years. The geographical centres of the CTs are shown in Figure 1B. The spatial resolution of these raw data was then determined by CT size, which is fixed based on the number of inhabitants or households in each region (Figure 1B).

**Figure 2:**
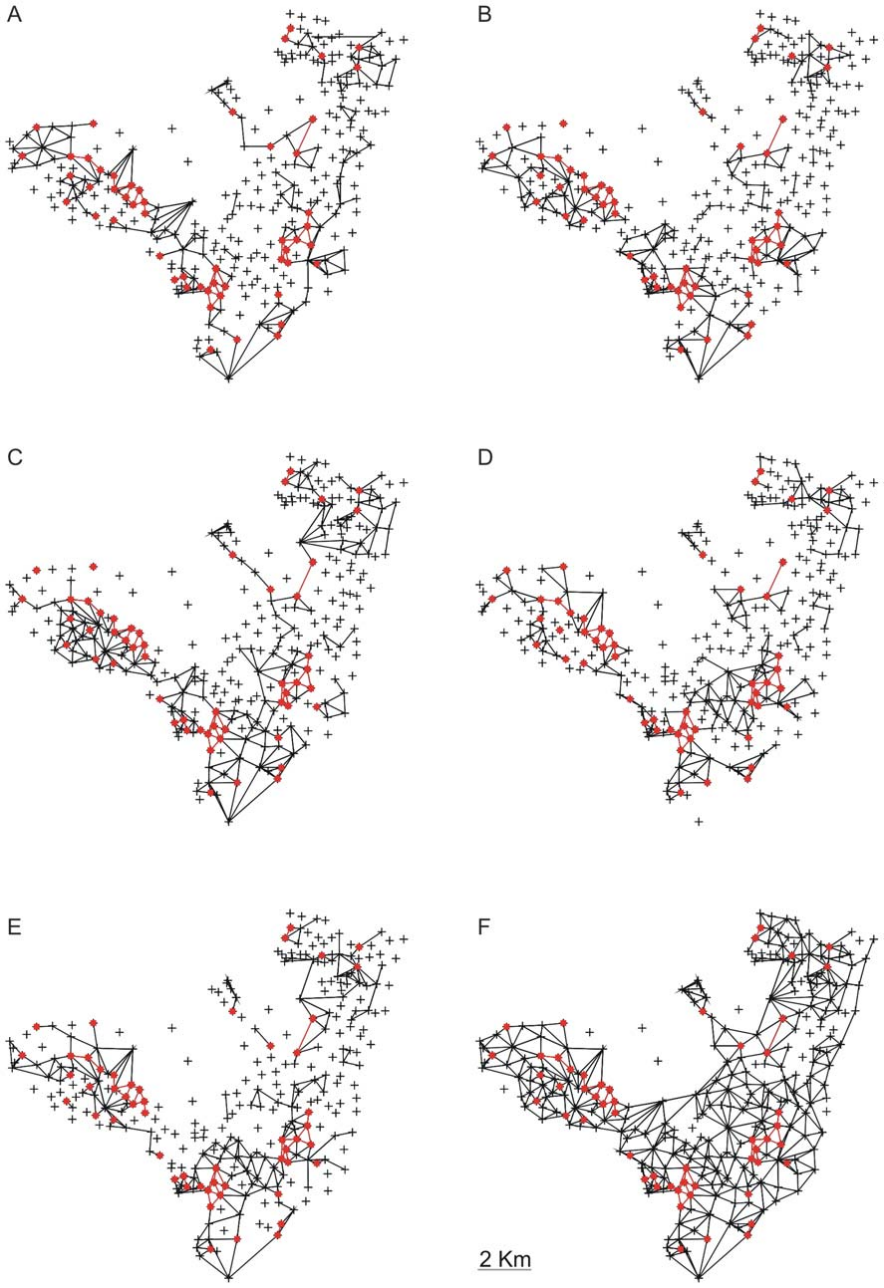
The annual path of the disease and foci CTs. In (A) 1996, (B) 1997, (C) 1998, (D) 1999, (E) and 2000 was obtained from the annual distribution of connectivities. The high-burden CTs and the links among them are highlighted in red and belong to all of the annual paths. (F) The epidemiological network (generated by the accumulation of the annual paths) within which disease dissemination takes place. From the mathematical point of view [16], this network can be studied with respect to its distribution of connectivities among the nodes (CT geometrical centres) and with respect to its ability to generate clustering and other effects that may influence the dynamics of disease spreading.

We based our tracing of the annual path of the etiologic agent on the annual distribution of cases (Figure 2A–E) by connecting all neighbouring CTs that presented at least one case of TB. The cases were basically distributed along one of two routes (South to Northwest, or South to Northeast), which correspond to the most heavily populated areas of the town. Although the distributions were not fully connected for all years in both directions, the dissemination of the disease continued to follow these paths despite these episodic discontinuities. Inspired by the concepts of percolation theory and dynamic propagation of information [16], we sought to identify a CT structure within the annual path sequences (Figure 2) that could guarantee the reproduction of similar connectivity distributions across the years analysed. Considering that the average time required for disease outcome (cure or fatality) is five years for complicated cases [17], we identified this structure by collecting the CTs that presented at least one new case of pulmonary TB in each of the five consecutive years. Actually the five-year estimate is based on a relatively universal old assessment and it would be useful to collect new data to reassess this estimate considering its possible variation with space and time.

**Figure 3:**
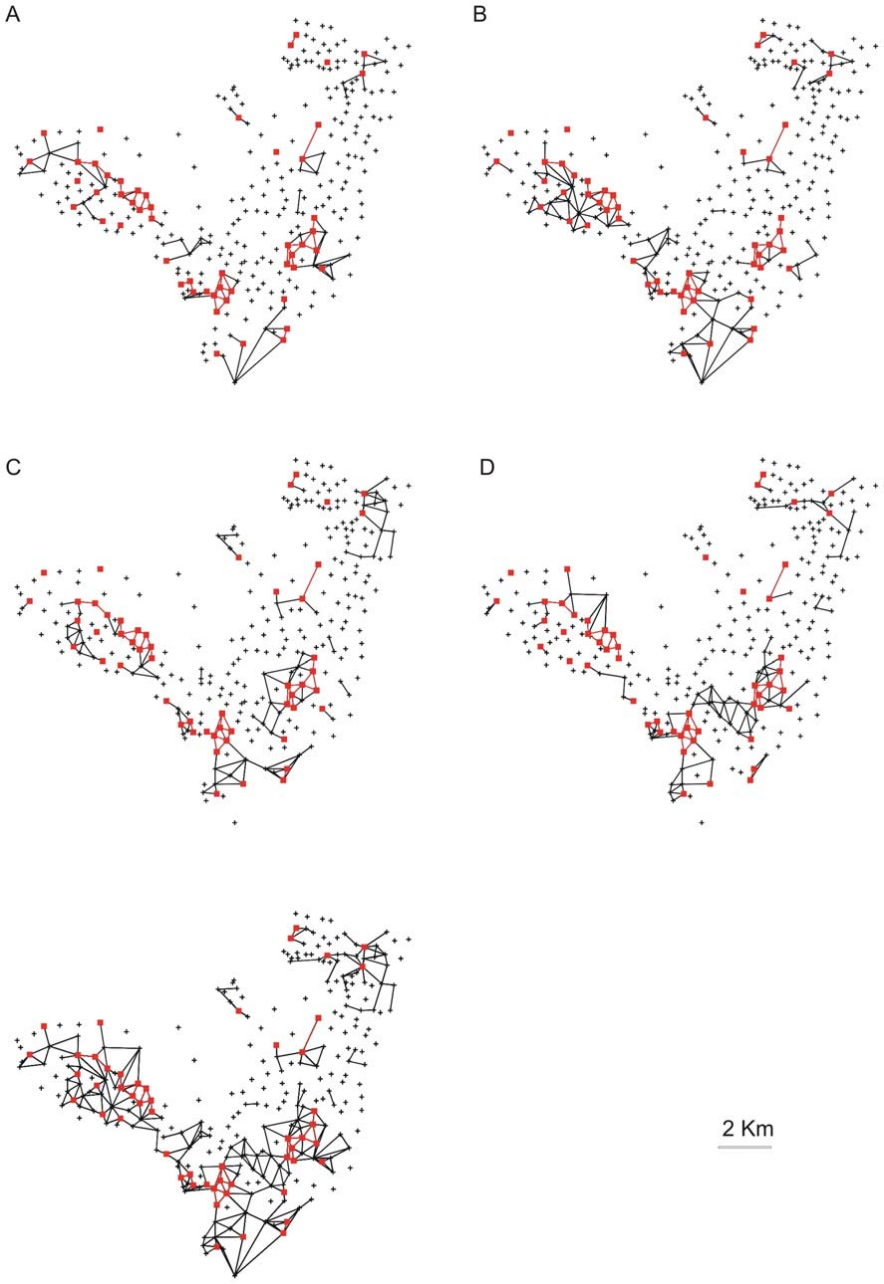
Identifying the CTs that maintain the foci. The sequence of maps shows, in black, the annual connections among the centres of the census tracts reporting infected patients for two consecutive years: (A) 1996–1997, (B) 1997–1998, (C) 1998–1999, and (D) 1999–2000. The 53 high-burden CTs that are sources of infections and the connectivities among them are highlighted in red.

**Figure 4:**
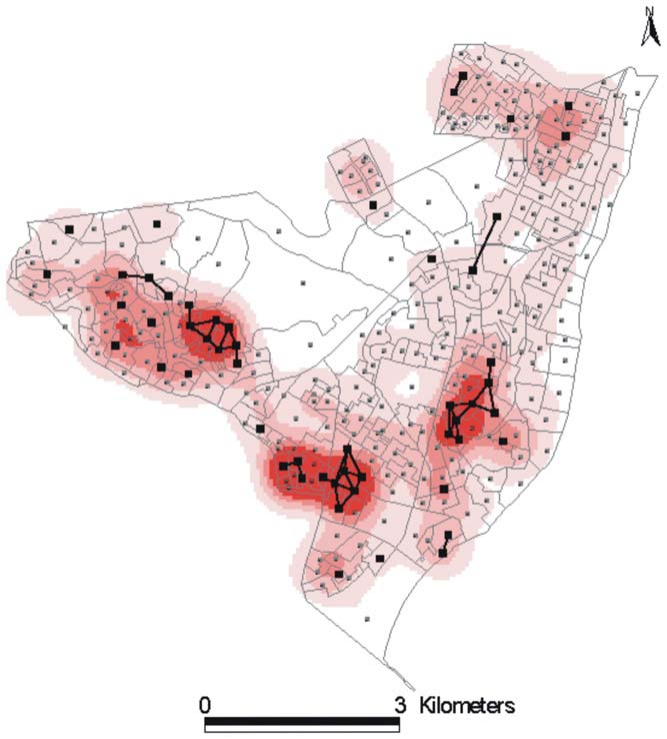
The role of high density household on the endemic process. The superposition of the household density distribution [13] obtained from the Census of 2000 and the network shown in Figure 3E. It becomes clear that the high density household favour the rapid transmission and manifestation of the disease, facilitating the communication between foci and their feeding.

We considered these high-burden CTs to be centres of activity of the disease. Thus, by identifying these foci in terms of a clinical criterion (time required for fatality or cure), we eliminated the need to adopt arbitrary criteria related to the social and environmental constraints implicit in the CT divisions. Among the 299 CTs in Olinda, we found 53 high-burden CTs (Figures 2, 3, 4) that should be kept under close surveillance and strict control, each one exhibiting on average more than 10 cases accumulated during the period.

Other criteria for selecting potential sources of infection are often adopted in the literature. In a previous work employing stationary methods [18], 77 CTs were considered to be high-burden CTs. In this work [18], the authors looked for CTs with a relatively high rate of transmission by cohabitants (e.g., more than one case per family) and/or CTs with re-treatment cases, which reflect treatment abandonment or drug resistance. Of the 53 focal CTs identified by our method only 31 were common to the 77 CTs previously reported [18]. In the same work, the authors classified 30 CTs whose mean incidence was above the 90^th^ percentile of the mean incidence distribution as high risk [18]. Our method revealed that only 21 of those 30 CTs were among the 53 high-burden CTs involved in the endemic process. Therefore, using this new, dynamic approach, based on the time correlations of raw data, an additional 32 previously undetected high-risk CTs were identified. Finally, we observed that 48 out of the 53 focal CTs have an accumulated number of cases that is equal to or greater than 1% of the average population of the CT and implies a relative risk equal to or greater than 2 (see Table S1). In other words, the 53 high-burden CTs identified by this novel methodology not only combined different conditions usually considered separately by stationary methods but also reflected the effects of the dynamics that maintained the infection during the CT's evaluated period.

A close inspection of socio-economic variables from the 2000 Census [15] showed that 90% of the 53 high-burden CTs identified were located in areas inhabited by individuals reporting the lowest levels of income and education (not shown). In addition, in the focal CTs, the number of households headed by women with less than one year of schooling and women who earned less than a minimum wage was on average 70–80% higher than the average in the low-burden CTs (Table 1).

**Table 1:** Average of some social, economic and environmental indices computed for regular and foci CTs.

Indices	Average of people/foci CT	Average of people/regular CT	Increase in foci CTs/regular CTs (%)	Total average of people/CT	Number of CTs with data recorded*
Females head of family with more than ½ and less than 1 minimum wage of income	57.3	32.1	78.5	36.6	299
Females head of family with income les or equal to ½ minimum wage	5.9	3.3	78.8	3.9	208(44)
Females head of family with one year schooling	9.8	5.8	70.7	6.5	268(52)
Females head of family with one year or less of schooling (including illiterates)	26.3	15.0	75.3	17.1	284
Females head of family illiterate (no schooling)	31.7	17.6	80.1	20.2	282
Head of family (male and female) without or with less than one year of schooling	49.8	30.2	64.9	33.8	290
Females head of family without schooling	22.2	15.1	47	16.4	288 (52)
% of cohabitants without instruction	14.2	9.6	48	10.4	299
% of cohabitants with income less or equal to one minimum wage	32.7	24.7	32.4	23.9	299
Females head of family	134	109	22.9	114	299
Average population size per CT	1429	1188	20	1230	299
Females head of family without income	222	198	12	202	299
Average number of cohabitants per household	3.94	3.77	4.5	3.80	299

The indices are presented in the same way (% or not) as they were collected during the 2000 Census. From the available information, no differences were observed between regular and high-burden CTs with respect to the presence or absence of piped water, toilets, and designated places for bulk disposure. However, the average population size per CT and women head of family is 20% greater in the foci than in the regular CTs.

*The total number of CTs was 299; however, the total average is taken from the total number of CTs for which the information was collected; that is indicated on the last column. We indicate in parentheses the number of foci for which there was information available, i.e. the total number of foci considered on the calculus of the average when it was different than 53.

We compared the following information for the 20 CTs that did not present any case during the period to the 279 that did present cases: percentage of heads of family with less than one year or no schooling, households with heads of family with salary less than or equal to one minimum wage, demographic density per Km^2^, average number of people per household at the CT, and average population size per CT. The Mann-Whitney test used to compare the differences between the two groups indicate that the percentage of head of family with less than one year or no schooling (p = 0.037), average number of people per household (p<0.001) and average population size per CT ( p = 0.002) are higher in the CTs that presented cases than in those that did not.

From Figure 2, it is clear that annual paths maintained the connections among the sources, which led us to inquire which CTs are responsible for sustaining the repeated infections in the foci CTs. In order to answer this question, we also took into account the characteristics of the clinical evolution of TB. Among infected individuals, only 10% develop the disease, and, among those, the disease appears within one year in 50%, within three years in 30%, and at some later point during their lifetime in the remaining 20% of cases [19]–[21]. Therefore, we considered the 50% of infected persons who develop the disease within one year to be those who play a key role in the local dynamics of rapid transmission of TB. In Figure 3, we show the connectivity distribution between the neighbouring CTs presenting newly reported cases for two consecutive years, which we believe are responsible for the rapid disease transmission and the maintenance of the high-burden CTs. In Figure 3E, we show the superposition of the paths that connect these CTs along the five years analysed. For each CT within this network of rapid transmission, the accumulated number of cases during the period (>5 cases) was greater than the average number of accumulated cases (3.4) for all 299 CTs. In other words, the method indicates another set of CTs that should be kept under close surveillance in addition to the 53 high-burden CTs. The additional information gained from the study of the dynamics of TB spreading would allow intervention in the persistence of the infection at the foci. Moreover, since many portions of this network are embedded in regions that have households with the highest reported densities of inhabitants (see Figure 4), the two-year correlations among new cases indicate that high household densities, in which there is close and prolonged contact, favour the rapid local transmission of TB. These results confirm that the household density is very important in determining the spread of infectious diseases, as noted in recent studies [10].

**Figure 5:**
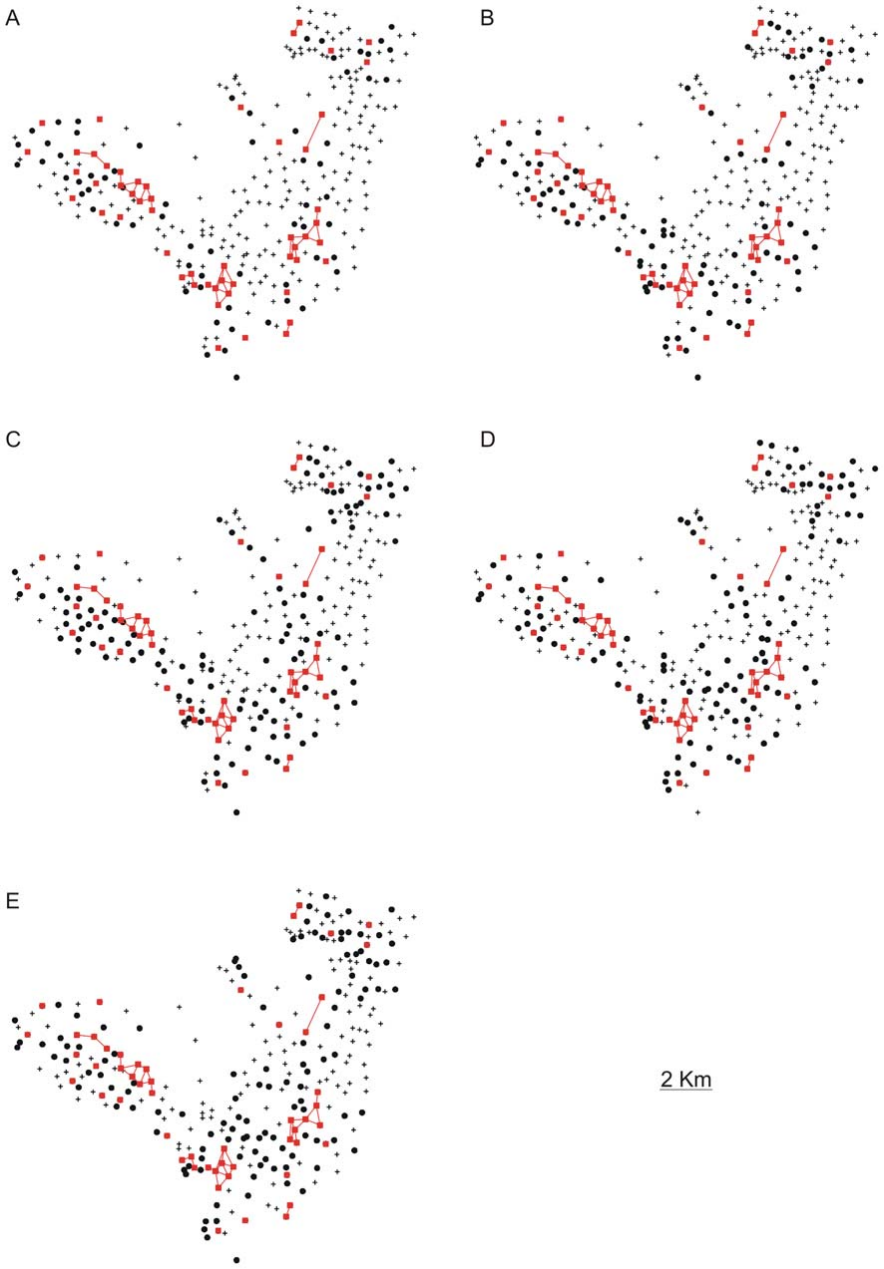
The disease moving among the neighbouring CTs. The sequence of maps corresponds to snapshots of Supplementary Movie S1, which shows the movement of the disease between the main regions that concentrate a large number of foci CTs. The black circles in each map correspond to the CTs with newly infected individuals that were neighbours of the CTs that had new cases in the previous year.

Finally, we used our data to analyse the dynamic aspects of TB spreading in Olinda. Towards that end, we traced the movement of the disease by identifying in a given year the CTs reporting new cases that were the nearest neighbours of CTs that presented new cases in the previous year. Movie S1 clearly shows that these CTs are fully connected along the five years analysed, as a result of the movement of new TB cases between sources. The details of this movement are shown in snapshots of (Figure 5). We observed that, initially, most new cases were located around the sources (Figure 5A). This initial pattern then evolves into subsequent patterns, which fully connect the high-burden CTs, and the final stage is reached by alternating between configurations that are all variations of the same kind of pattern. The overall movement looks like a wave that oscillates between the identified sources of infection reflecting a mechanism that feeds back upon them. This shows that the sources that our approach identifies constitute the backbone of the disease transmission, supporting the main assumptions of our method and showing its relevance. As Movie S2 demonstrates, another mechanism can be observed that shows the continuous presence of the disease around the 53 focal neighbourhoods. In order to arrest the proliferation of the disease, this maintenance in focal CTs must be interrupted. By cross-referencing these data with those related to the network of rapid transmission of the disease, it is possible to accurately target surveillance and control in the key CTs, in order to prevent the occurrence of new TB incidents. This strategy will help reduce the rates of local TB incidence and mortality in this town. Based on the data that is currently available we can only relate regions of high TB incidence with household density. It would be interesting to have additional information, particularly, on work, leisure and how these people move in town, to be able to connect our findings to other places where people repeatedly spend time and transmission could occur.

**Figure 6:**
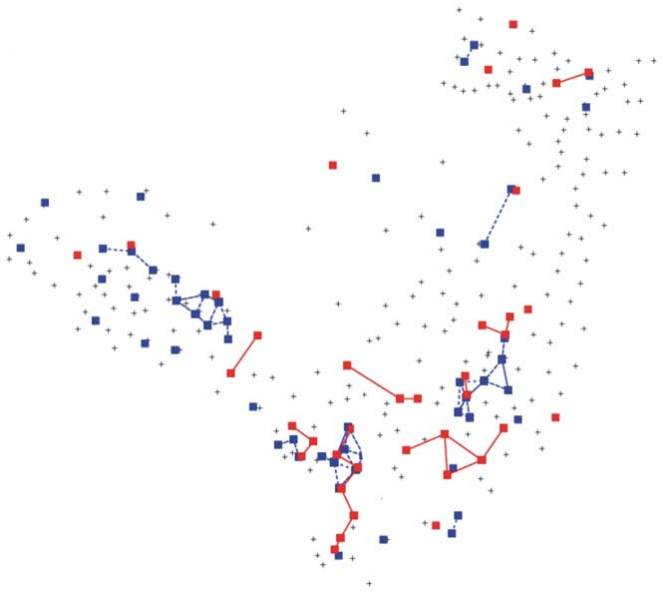
Comparison of the position of the high burden CTs in two different periods. Using the GC distribution map for the period of 1996–2000 the 53 foci were marked in blue, while in red was indicated the position of the 37 foci from 1991–1995. Therefore in this figure the red foci do not correspond to the GCs of the 1996–2000 period.

In fact the data we had access to was composed of two sets of five consecutive years of annually geo-referenced data. One corresponds to the period of 1991–1995 that was geo-referenced to 243 census tracts of Olinda from the 1990 Brazilian Census and the other corresponds to the period 1996–2000 that was geo-referenced to 299 census tracts from the 2000 Brazilian Census. Because of the different number of census tracts, the data corresponding to both periods could not be pooled together. Here we describe in detail only the analysis of the later period since the results for both periods are rather similar. However we have also applied the same methodology to analyse the data corresponding to the period from 1991 to 1995. For this period the method pointed out 37 CT s as sources of infection. Since the 243 CTs of the 1990 Census were redefined into new 299 CTs in 2000 Census, in Figure 6 we superpose the two maps and show in red the 37 sources of infection during the period 1991–1995 and in blue the 53 from the period of 1996–2000. Since the correspondence between the different CTs division can not be recovered, the fact that the total number of CTs have increased 20% from one period to another justify the apparent increase in the number of foci. The important feature to be pointed out is that the CTs foci remain in the same region in both periods and the results from the analysis of the two periods are similar as it should be expected in order to validate the approach.

## Discussion

The method we have presented here is easily implemented and provides relevant information concerning the spatio-temporal propagation of TB using GIS data. In comparison to more traditional methods, our approach is novel for the following reasons. 1) Instead of showing high-risk regions, it precisely locates the main sources of infection in the high risk regions determined by the usual statistical approaches. 2) Different from the usual statistical approaches that filter and transform the raw data to generate risk or probability density functions, it allows to extract relevant information directly from the raw data, as for instance the movement of the etiological agent. Because of this, the technique presented here, unlike stationary ones [6], [7], is not sensitive to the irregular distribution of CTs. 3) Since this method is based on the study of individual settings (a town or state) it avoids generalizations that might overlook the specificities of TB transmission in different communities and regions. 4) Finally, our method is not only descriptive but is also useful for evaluation purposes because the analysis of the case distribution after the implementation of any control strategy would provide information on the strategy's efficiency. The changes could be evaluated by comparing a series of quantities, among them, the number of foci, the size (both in terms of the number of links and of the spatial area) of the largest connected cluster of neighbour CT's with at least one new case per year, etc. In all cases, changes in these quantities should be compared during several years in order to discard fluctuations.

By taking into account only local interactions, the method is able to identify the backbone of the disease transmission showing that household density is a key factor which suggests that a continuous and prolonged exposure to other infected people is necessary to become infected.

In addition, our results show that programs of surveillance and control need to be based on the study of the dynamics of the spatial distribution of cases [4], [5], [22] so that they might best identify the sources as well as the feedback mechanisms that sustain the infectious process. Furthermore, the results depicted in Table 1 indicate that poverty, limited schooling, and poor hygiene habits apparently reduced the quality of life of the families with the largest incidence rates in ways that favoured the transmission of TB, as well as many other diseases [19]. These results indicate that in order to target these sources efficiently, the disease control strategy should involve not only the Directly Observed Treatment, Short-course Program (DOTS) [20], and specific territorial control, but also specific educational programs that improve the quality of life for those families, thereby decreasing the rates of treatment abandonment and reducing the numbers of cases within individual households.

The simplicity of the method presented here, and the advantages of its results compared to stationary methodologies, provide indications for how to interrupt disease transmission in ways never considered before. By identifying the truly important CTs that should be kept under surveillance, this method would also help to scale up the DOTS program, guide budget appropriations, and efficiently allocate skilled human and diagnostic resources, thereby facilitating the rapid identification of new cases and vaccination schedules. This approach would be also very useful to implement the DOTS Plus Programs [23] for the control of multi-drug resistance-TB cases (MDR-TB). The rise of MDR-TB calls for rapid adoption of new public health strategies that aim at reducing drastically the (currently increasing) rate of new incidences. Using the approach introduced here to understand the MDR-TB spatio-temporal dynamics and its role on the overall TB spreading will help the design of such strategies.

Essential information for disease control can be obtained by applying this method to long-term contemporary cohort studies that include data from sputum smear microscopy for identification of the bacterial strain by acid-fast bacilli culture, and from family-based association analysis, together with a linkage study that involves relatives. For example, this method can provide insight into the dynamics of the appearance of new bacterial strains or into the number of secondary cases generated by one infected individual. Finally, this method can be applied to the epidemiology of other infectious diseases, as well as chronic diseases, as long as the specifics of the disease in question are taken into account in each case. An application of this approach to the study of vector-borne diseases would have to include the spatio-temporal correlations in the concentrations of vectors and infected individuals. This method can also be successfully applied to a dynamic analysis of other social issues, such as increased violence or variations in socio-economic factors.

## Methods

### Data acquisition

The data analysed here correspond to the new pulmonary TB cases reported annually to the National Disease Notification System of the Ministry of Health (Sistema de Informação de Agravos de Notificação, SINAN). The data do not distinguish between contact transmission and latent TB reactivation. Recent results suggest that latent TB reactivation comprises a small proportion of the cases [24], therefore, in our approach all cases are considered as if they were generated by recent contact transmission. The approval of an ethics committee was not needed because the project was developed using secondary data from the public health care system provided by the local health authority, with the author's commitment to ensure data privacy. Each TB case was geo-referenced with respect to the 299 census tracts (CTs) into which the town of Olinda was divided by the Brazilian Census of 2000 (Instituto Brasileiro de Geografia e Estatística, IBGE) [15]. The CT localization of each case is chosen based on the home address of the infected person. The geo-referenced data correspond to 85% of the reported cases. The Census of 2000 also provides detailed information about social, economic and health indices, which were used to analyse our results.

### Pre-processing of the data and main definitions

Using the information provided by the 2000 Census we are able to reproduce the distribution of geographic centres (GC) computationally. We define as neighbour CTs those that share a common polygon side. Other definitions like setting as neighbour CTs those belonging to a circular area of 300m radius were also considered but proved to generate equivalent results. The 300 m radius was chosen based on the facts that temperature in Olinda is usually high (annual average ∼29 C) and the city is built on an irregular terrain with many uphill roads which favour people to do business and stay close to their own homes. Once the spatial distribution of GC and its neighbourhood is defined, for each year (t = 1996, 2000) we associate the number of accumulated new TB cases of a given 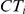, 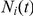 (i = 1,299), to the point that represents its GC. These distributions describe the raw geo-referenced data we had access to and would be subjected to a detailed analysis.

In order to determine the annual aetiological agent path, we connect with segments the centres of neighbouring census tracts that given a year present at least one infected patient. In other words for each year (t = 1996, 2000) we connect the neighbouring sites *i* and *j* if 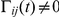 where 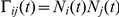. We refer to these segments as links and to the collection of links for each year as the annual distribution of connectivities. In Figure 2 we show these distributions for the five years as well as the network composed by its superposition showing the crosslink between them that favour the spread of disease. Observing the five year paths we realize that the disease has percolated in the town.

### Motivation for the analysis and major assumptions

Percolation theory [25] was first used to describe the polymerization process that may lead to gelation: how small branching molecules form large macromolecules that ultimately would form a network spanning the whole system. The sequence of paths shows that the information (in this case TB) has spanned Olinda during the entire period. Therefore the key point in this study is to find out the common structure of CTs in those 5 paths that guarantees that the percolation will be maintained during the five years. Mathematically, this structure would be determined by removing the dangling bonds (*i.e.* “loose ends”) along each path and choosing the common structure that would be present in all five annual paths. This percolation patterns emerge, however, from social processes and by removing the dangling bonds we would mask and interfere on the information to be extracted from this analysis. Hence, we have used the characteristic times related to the evolution of the disease to search for the CT or structure of CTs that are sources of infection and the CTs that are responsible to maintain (or feed) these sources. The characteristic times were obtained from the estimates that only 10% of infected people develop the disease: 5% in one year, 3% in three years and 2% during their lifetime; and that for the complicated cases after 5 years either the patient is cured or diseased.

In order to define the nodes and the links of the network that we use in our method we have assumed that all CT's have the same properties even though they span different geographical areas. Consequently, we do not distinguish among them *a priori* according to population density. If there is any distinction, it comes from the subsequent analysis. We do not consider any other possible heterogeneity, particularly, in people behaviour. As mentioned before, we assume that people tend to stay within a restricted area for most of their daily activities. For this reason, we assume that the disease propagates through local interactions.

### Data analysis

Since five years is the average time necessary for reaching an outcome (death or cure) in complicated cases of TB [15], [19], we define any 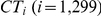 that presents new cases during five consecutive years as a focus or source of infection. Therefore, a focus is a 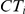 for which the product of 
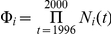
 is different from zero, being *N_i_*(*t*) the number of the yearly new reported cases during the year *t*.

Once we have located the high burden CTs (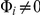) which continuously produce new cases, we search for those CTs that sustain the infection on these sources, looking for the cases that evolve rapidly to the disease. Hence, we looked for those that develop the disease in one year, searching for the 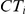
s that have had at least one case during two consecutive years, or 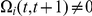, where 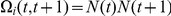 and t = 1996, 1999. The distribution of connectivities for each pair of years (Figure 3, showing in red the 53 high burden CTs) is built between the neighbouring CTs *i* and *j* for which 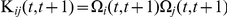 is different from zero. The small sub network generated by these CTs is embedded in the high density household region, showing that these regions actually are responsible for the maintenance of the high burden CTs (Figure 4).

In order to study the movement of the disease (Figure 5 and Supplementary Movie S1), we identified all CTs presenting at least one new case during the current year (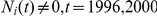) and having at least one neighbour presenting one or more new cases during the previous year (*t*−1). In other words, we select for each year all 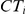
s for which the quantity 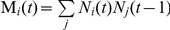 is different from zero, with the sum over *j* taking into account all the *j* neighbours of the CT *i*.

The movements of the disease shown in Movie S2 is obtained by identifying for each year the CTs that are nearest neighbours of the 53 high-burden CTs and have at least one new case. In other words we show the dynamics of new cases in the neighbourhood of the sources of infection.

## Supporting Information

Table S1 Average annual TB incidence rate in the city of Olinda, Brazil. The number of cases and the population is presented annually for the entire period of 1996–2000. The average incidence rate per 100,000 inhabitants of each year is also shown and the average incidence rate during a five-year period is also calculated.
(0.03 MB DOC)

Movie S1 The dynamics of TB spread in Olinda. The movement of TB between the foci (red squares) was traced by identifying in a given year the CTs with new cases (black circles) that are nearest-neighbours of the CTs that had new cases in the previous year. The movie shows that the annual movement of the new TB cases keep the high-burden CTs fully connected along the five years analysed. Initially, most of the new cases are located around the sources. This first pattern evolves into subsequent ones that fully connect the foci CTs. This movement reflects a feedback mechanism for the high-burden CTs in the period analysed.
(0.16 MB MPG)

Movie S2 Movement of new TB cases around the 53 high-burden CTs. These movement was obtained identifying the CTs (black dots) with new TB cases that were neighbors of the foci CTs (red squares) for each year.
(0.14 MB AVI)
