# Supplementary material for: A Dynamic Analysis of Tuberculosis Dissemination to Improve Control and Surveillance
Source: PLoS One. 2010 Nov 30;5(11):e14140. doi: 10.1371/journal.pone.0014140 (PMC2994743; doi:10.1371/journal.pone.0014140)
Supplement: Table S1 — Average annual TB incidence rate in the city of Olinda, Brazil. The number of cases and the population is presented annually for the entire period of 1996–2000. The average incidence rate per 100,000 inhabitants of each year is also shown and the average incidence rate during a five-year period is also calculated. (0.03 MB DOC) [file pone.0014140.s001.doc]

**Table S1. Average annual TB incidence rate in the city of Olinda, Brazil.**

| Year | Number of cases | Population | Incidence rate per 100000 inhabitants |
| --- | --- | --- | --- |
| 1996 | 433 | 349243 | 124.0 |
| 1997 | 411 | 353783 | 116.2 |
| 1998 | 419 | 358381 | 116.9 |
| 1999 | 381 | 363039 | 104.9 |
| 2000 | 340 | 367758 | 92.5 |
| Total Number of TB cases* | 1984 |  | 110.7** |

* Annual Average of TB cases 396.8

** Average Incidence Rate per 100,000 inhabitants
